# Conservative Management in Congenital Bilateral Upper Eyelid Eversion

**DOI:** 10.1155/2015/389289

**Published:** 2015-04-15

**Authors:** Viola Andin Dohvoma, Alice Nchifor, Aronette Nana Ngwanou, Elisabeth Attha, Faustin Ngounou, Assumpta Lucienne Bella, Côme Ebana Mvogo

**Affiliations:** ^1^Faculty of Medicine and Biomedical Sciences, University of Yaoundé I, Yaoundé, Cameroon; ^2^Presbyterian Eye Clinic Acha, Douala, Cameroon

## Abstract

*Aim*. To report the case of congenital bilateral upper eyelid eversion with severe chemosis that was successfully managed conservatively. *Report*. The patient was a six-hour-old male neonate with bilateral congenital upper eyelid eversion and severe chemosis, following uneventful delivery. Conservative management consisted of the application of antibiotic ointment and padding the exposed conjunctiva with 5% hypertonic saline-soaked gauze. The eyelids reverted spontaneously on day 3 and the condition was completely resolved by the third week. *Conclusion*. Congenital upper lid eversion is usually a benign condition which responds well to conservative treatment. Creating awareness amongst healthcare professionals is essential.

## 1. Introduction

Congenital eyelid eversion is a rare condition in which the eyelid is completely turned out, with prolapsed conjunctiva and chemosis. Usually, cases present at birth, although late presentations have been reported [1, 2]. It is typically a bilateral condition.

The exact cause is unknown. Most cases reported have no associated ocular or general abnormalities. The condition is, however, more frequently seen in black infants, in Down syndrome, and in collodion skin disease [1, 3].

Most cases are treated medically. Surgical management is reserved for cases with late presentations or with complications or cases not responding to medical management.

Two cases have so far been reported in Cameroon. Management in one case consisted of manual repositioning of the tarsal conjunctivae and bandaging of the eyelids following antibiotic ointment application [4]. In the other case, multiple punctures of the conjunctiva were made [5].

We report a case of bilateral upper eyelid eversion with severe chemosis that was successfully managed conservatively leading to the spontaneous inversion of the eyelids.

## 2. Case Presentation

A six-hour-old male newborn was referred to our eye clinic from a private hospital in which he was born for the management of the eversion of both upper eyelids. He was the sixth child to a 29-year-old woman. A history of malaria in the third trimester was reported. This was managed accordingly. Pregnancy was regularly followed up. Delivery was at term. Labour and delivery were both uneventful.

**Figure 1:**
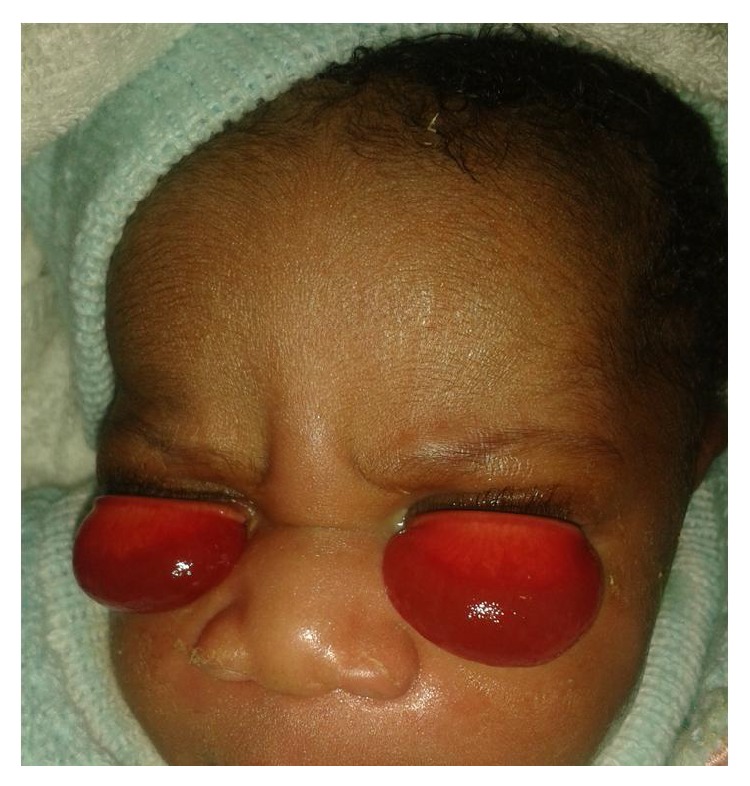
Six-hour-old newborn with upper eyelids everted and severe chemosis.

Examination revealed bilaterally everted upper eyelids with severe chemosis (Figure 1). Examination after instillation of a topical anesthetic agent and retraction of the upper eyelids with Desmarres retractors revealed grossly normal eyeballs with negative fluorescein staining of the cornea in an otherwise healthy looking baby.

The baby was admitted and managed as follows: application of neomycin and polymyxin eye ointment and padding of the prolapsed conjunctiva with gauze dressings soaked in 5% hypertonic saline once daily. Daily eye cleansing was performed to keep the external eye clean of any discharge.

**Figure 2:**
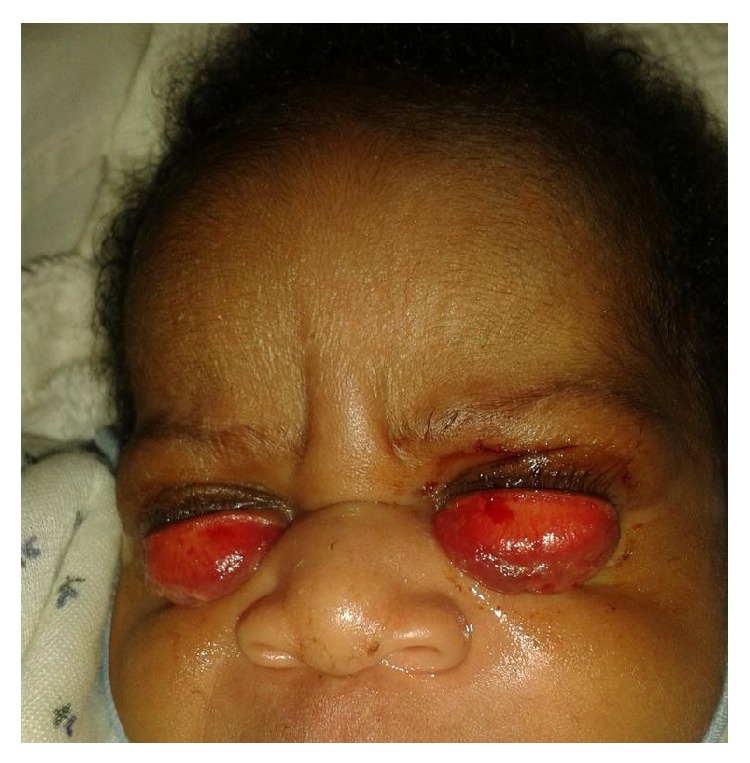
Significant reduction in chemosis on day 2.

**Figure 3:**
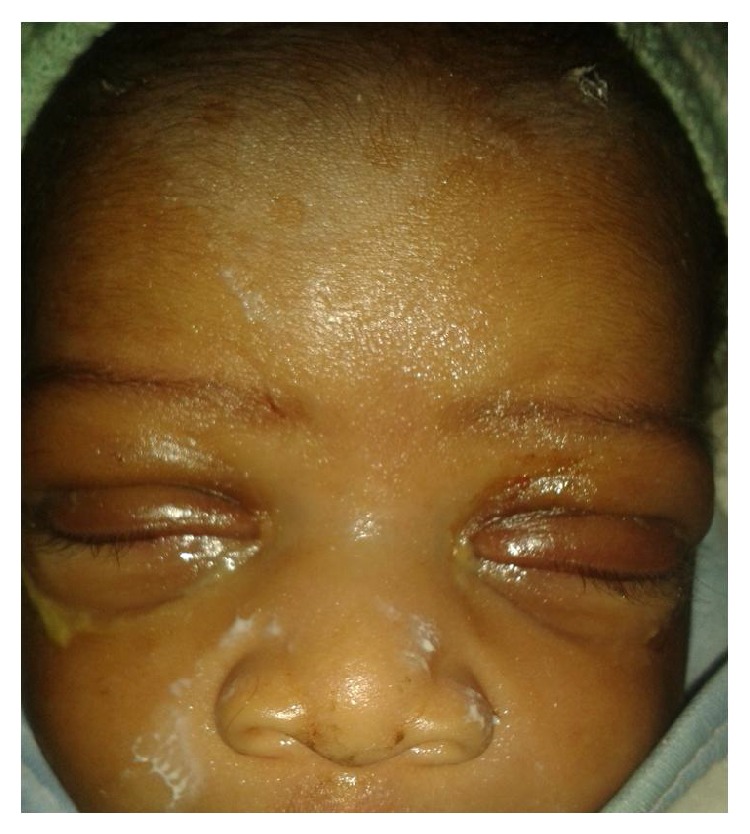
Spontaneously reverted eyelids on day 3 with mild oedema present.

On the second day, there was reduction in the size of the chemosis (Figure 2). Treatment was continued. On the third day, the chemosis fully resolved and the lids reverted back to normal position (Figure 3) but everted mildly at the margin when the baby cried. The baby developed fever with a temperature of 39°C. Neonatal sepsis was suspected and the baby referred to the neonatal unit of another hospital with an available ophthalmic unit.

Paediatrician examination revealed no morphological abnormality. C reactive protein was positive at 12 mg/L. Full blood count showed white cell count in the normal range but the differential count revealed granulocytosis. Urine and cerebrospinal fluid cultures were negative. The baby was admitted and treated for neonatal sepsis with intravenous Ampicillin, Netilmicin, and Cefotaxime for 8 days. While being admitted, the eyes were no longer padded and 5% hypertonic saline eye drops were administered four times daily; antibiotic ointment was used twice daily. Fever subsided on the 5th day.

**Figure 4:**
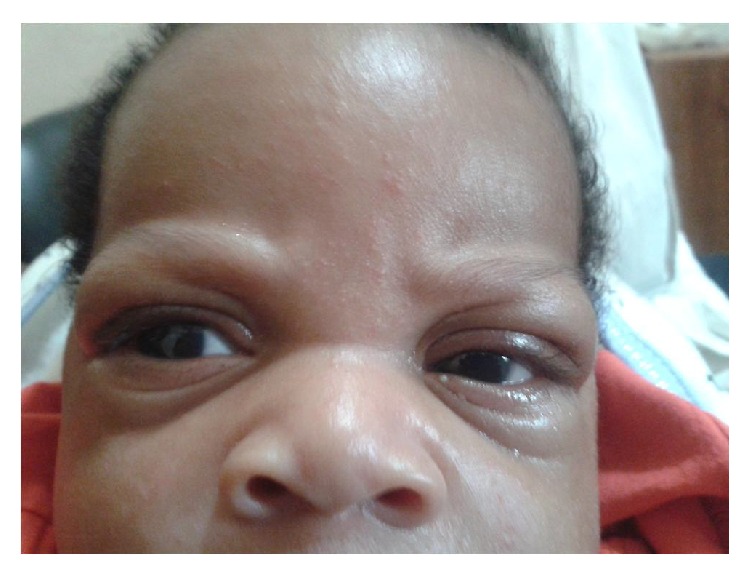
Spontaneous opening of the eyes on the third week.

At 3-week followup, the child could open the eyes spontaneously (Figure 4). Ocular examination revealed clear corneas; anterior chambers of normal depth; round, central, and reactive pupils; and normal fundi.

## 3. Discussion

Complete eversion of the upper eyelids with chemosis is a rare condition. Sellar et al. [1] in 1992 reviewed the literature and found 51 cases reported. There is no proven aetiology of the condition. Several possible mechanisms have been proposed such as vertical shortening of the anterior lamellar or vertical elongation of the posterior lamellar of the eyelid and failure of the orbital septum to fuse with the levator aponeurosis (with adipose tissue interposition) [6].

Congenital eyelid eversion can be treated conservatively. The goal of management is to prevent desiccation of the exposed conjunctiva and allow spontaneous inversion of the lid. Conservative management includes applying moist dressings, eyelid taping, and pressure patching along with topical antibiotic and lubricants. Additionally, topical 5% hypertonic saline has been employed as a conservative treatment by some authors [7, 8]. The mechanism by which the 5% hypertonic saline-soaked gauze dressing worked as explained by Adeoti et al. [7] is the osmosis of fluid from the oedematous tissues through the semipermeable subconjunctival membrane.

Surgical treatment options reported in the literature include scarification of the exposed conjunctivae [5], temporary tarsorrhaphy [9, 10], subconjunctival injection of hyaluronic acid [11], fornix sutures [11], full thickness skin graft to the upper lid [12], and compression eyelid sutures [13].

Our case was managed using topical antibiotic ointment, to avoid infection and drying of the exposed conjunctiva, padding with 5% hypertonic saline soaked gauze to reduce chemosis by osmosis. No manual manipulation of the lids was done. Improvement was observed within 3 days and complete resolution occurred in 3 weeks with no sequelae. Our case had neonatal sepsis. In a report on three cases, Adeoti et al. [7] reported that one of the patients had manifestations of neonatal sepsis.

Lid manipulations can lead to autonomic effects such as respiratory arrest in neonates. This has been reported by Watts and Dapling [14]. This further advocates the need for a strictly conservative approach in management.

If not treated early, it could lead to complications like secondary infections and epidermization of the conjunctiva. The Chemotic conjunctiva protects the cornea from exposure and hence, corneal complications are rare. However, a case of corneal perforation has been reported in a baby with Down syndrome [12].

The presentation of congenital eyelid eversion is usually alarming to both the parents and the healthcare professional, especially if he/she is seeing it for the first time. Its benign course, however, as shown in this case, justifies a conservative approach in anticipation of an excellent result. It is important to create awareness among healthcare professionals in obstetric and neonatal care of the existence of this congenital anomaly, as the condition responds well to early treatment.
